# Temporal Associations Between Body Checking and Eating Pathology in Adolescent Girls with Binge-Spectrum Eating Disorders: Protocol for an Ecological Momentary Assessment Study

**DOI:** 10.2196/73447

**Published:** 2026-04-15

**Authors:** Jannah Moussaoui, Stephanie Manasse, Laura D'Adamo

**Affiliations:** 1 Center for Weight, Eating, and Lifestyle Science, Drexel University, Philadelphia, PA, United States; 2 Department of Psychological and Brain Sciences, Washington University in St. Louis, 1 Brookings Dr, St. Louis, MO, 63130, United States, 1-314-935-5843; 3 Center for Healthcare Delivery Science, Nemours Children's Health, Wilmington, DE, United States; 4 School of Medicine, Washington University in St. Louis, St. Louis, MO, United States

**Keywords:** binge eating, adolescents, body checking, eating disorders, ecological momentary assessment

## Abstract

**Background:**

Up to 92% of individuals with eating disorders (EDs) report engaging in body checking behaviors (eg, repeated self-weighing and pinching of various body parts) to assess their weight and shape. These behaviors contribute to increased body dissatisfaction, negative affect, and dietary restriction, thereby maintaining ED symptomology.

**Objective:**

This study aims to first characterize the types and frequency of body checking behaviors (eg, self-weighing, pinching) reported among adolescent girls with binge-spectrum EDs during a 21-day ecological momentary assessment (EMA) protocol. The second aim is to explore the prospective associations between body checking and cognitive and behavioral ED symptoms, namely body dissatisfaction, fear of weight gain, dietary restraint, dietary restriction, compensatory behaviors, and binge eating. The third aim is to assess whether body checking behaviors show reactive effects (ie, produces change in the behavior subject to monitoring) to EMA, such that they decrease over time.

**Methods:**

The study will recruit 70 adolescent girls aged 14 to 19 years with clinically significant binge eating. Participants will complete a semistructured interview and a series of self-report measures at baseline to assess ED pathology. Then, participants will complete 5 daily EMA surveys to track body checking behaviors and related ED symptoms over 21 days.

**Results:**

Recruitment began in January 2025, with data collection expected to conclude in March 2026. Linear and generalized mixed-effects models will be used to evaluate concurrent and prospective associations between various ED symptoms.

**Conclusions:**

This study will provide insights into the patterns and impacts of body checking behaviors among adolescent girls with binge-spectrum EDs. If body checking behaviors reduce in response to EMA, digital self-monitoring could be a scalable and cost-effective strategy for ED treatment and prevention. The findings may also inform the development of momentary interventions targeting body checking behaviors to mitigate ED symptoms. Future research should extend these observations over longer periods and include male participants to generalize findings across genders.

## Introduction

### Background

Binge-spectrum eating disorders (EDs), including bulimia nervosa (BN) and binge eating disorder (BED), are serious psychological disorders characterized by recurrent episodes of binge eating (ie, eating an excessive amount of food while experiencing a sense of loss-of-control over one’s eating) and may be accompanied by behaviors intended to compensate for these episodes (eg, vomiting or restricting food intake). Binge-spectrum EDs typically start during adolescence [1] and have a 7% to 8% prevalence among adolescents [2,3]. Critically, gold-standard treatments for binge-spectrum EDs fail to produce remission in 60% to 75% of adolescents [4-6], and only about 50% of individuals stay remitted 5 years following treatment [7]. These low remission rates indicate that available treatments do not sufficiently address all mechanisms maintaining the ED. Indeed, following treatment, many continue to experience ED symptoms, despite no longer meeting diagnostic criteria for an ED [6,8]. In particular, body image disturbances remain elevated following treatment [9,10], and studies have identified elevated body image disturbances as a risk factor for relapse [11]. These findings are consistent with the transdiagnostic model of cognitive behavioral therapy (CBT) for EDs, which points to the overvaluation of weight, shape, eating, and their control as the core psychopathology underlying these disorders [12], thereby suggesting that until overvaluation is addressed, individuals may continue to experience ED symptoms. Given the low remission rates among adolescents with binge-spectrum EDs, it is critical to understand how the overvaluation of shape, weight, and eating manifests among adolescents and how it relates to ED symptomology.

One behavioral manifestation of overvaluation with weight, shape, eating, and their control is body checking [13]. Body checking refers to a broad range of behaviors used to assess one’s weight or shape (eg, self-weighing, checking how clothes fit, pinching or feeling various body parts) [14]. Based on studies among adults with EDs, these behaviors have a 90% prevalence [13,15]; are often engaged in compulsively; are brief, typically lasting less than two minutes [13]; and can occur multiple times in a given day. For example, in a transdiagnostic, qualitative study of adults with anorexia nervosa and BN, 92% of adults endorsed body checking and 33% of adults reported body checking 16 or more times in a given day [13]. Likewise, in 2 ecological momentary assessment (EMA; ie, an ecologically valid data sampling approach that prompts participants multiple times over the study period) studies among female college students with high weight and shape concern, participants body checked an average of 16 to 28 times a day [16,17]. However, no study has yet attempted to estimate how frequently body checking occurs among adolescents, let alone those with binge-spectrum EDs.

Critically, body checking behaviors are typically aimed toward disliked body parts [14], and selectively attending to disliked body parts and their perceived changes may contribute to increased ED cognitions, particularly body dissatisfaction and body image disturbances, and behaviors [18]. In transdiagnostic adult ED samples, body checking is associated with increased dietary restriction, meal skipping, use of fluids to curb appetite [19,20], increased negative affect [21,22], and shape and weight overconcern [15]; among those with binge-spectrum EDs, body checking is additionally indirectly tied to binge eating through increased body shame and appearance anxiety [23]. Further, 1 longitudinal study found that in a community sample of adolescents, higher body checking at 1 time point was associated with the development of ED symptoms 4 months later [24], underscoring the need for early and targeted interventions for body checking behaviors in this age group. Indeed, adolescents report high rates of body image dissatisfaction [25], a prominent driver of ED pathology among adolescents [26-28]. In recent years (ie, 2017‐2022), the prevalence of EDs has dramatically increased among adolescent female participants [29]. Despite this, few studies have examined the associations between body checking behaviors and ED symptomology among adolescents with binge-spectrum EDs.

Given the strong theoretical links between body checking behaviors and ED symptomology, body checking may, itself, be a worthwhile target for intervention. In fact, 2 EMA studies among female college students found small decreases in body checking behaviors as well as body dissatisfaction and internalization of the thin ideal. These reductions can be understood as effects of self-monitoring. In cognitive-behavioral models of psychopathology, the self-monitoring of behaviors and psychological states is key for reducing engagement in undesirable behaviors and cognitions [30], including body dissatisfaction [31], ruminative thinking [32], suicidal ideation [33], depressive symptoms [34], sedentary behaviors [35], and substance use [36]. Self-monitoring theoretically increases the awareness of the frequency, antecedents, and consequences of problematic thoughts and behaviors [30], allowing individuals to make changes either independently or with the support of a clinician. It is possible that during EMA, participants experience reductions in body checking frequency because reporting these behaviors on EMA surveys similarly increases the awareness for the frequency, antecedents, and consequences of these behaviors. Thus, in addition to examining associations between body-checking behaviors and ED symptomology in the real world, it is also worthwhile to investigate how these behaviors may decrease in response to self-monitoring. Notably, if body checking behaviors show reductions during EMA in adolescents with binge-spectrum EDs, and if these reductions are associated with improvements in other ED symptoms, then digital self-monitoring may be a powerful tool to formalize body checking as an intervention target in evidence-based treatments such as enhanced cognitive behavior therapy (CBT-E) or as a stand-alone treatment. Given the large ED treatment gap (eg, less than 20% of individuals with EDs receive care [37]), the scalability of digital self-monitoring could prove valuable among those engaging in body checking [16,17] prior to the receipt of more intensive treatment or as a prevention tool, particularly among adolescents.

### Present Study

Despite the relevance of body checking as a maintenance factor for ED pathology, relatively little is known about the nature of body checking and its prospective associations with ED cognitions and behaviors among adolescents with binge-spectrum EDs. Thus, this study aims to fill 3 specific gaps. The first aim of this study is to characterize the types and frequency of body checking behaviors among adolescent girls (ages 14‐19 years) with binge-spectrum EDs during a 3-week EMA protocol. Although body checking behaviors are present across the ED spectrum, they have most frequently been studied within restrictive EDs, despite the evidence of their presence within binge-spectrum EDs [13] and associations with binge eating [23]. Similarly, while body checking is prevalent among boys [24], consistent with other studies [16,17], we elected to restrict this study to girls because measures in this study would fail to capture the different behaviors used to body check and their associations with ED behaviors in boys [38,39]; further, resource constraints (ie, limited budget and personnel) precluded the recruitment of boys. We expect that participants will report a broad range of body checking behaviors and that they will engage in body checking behaviors several times a day on average. The second aim of the study is to test prospective associations between body checking and other ED symptoms. Consistent with the CBT model for EDs, we expect that body checking will be concurrently and prospectively associated with cognitive (ie, body dissatisfaction, fear of weight gain) and behavioral (ie, dietary restriction or restraint, compensatory behaviors) ED symptoms. The third aim is to assess whether the self-monitoring of body checking produces decreases in body checking over time. We hypothesize that body checking will decrease over the study period and that participants will report lower frequencies of body checking on the last 3 days of the protocol compared to the first 3 days [16,17].

## Methods

### Ethical Considerations

Prior to study enrollment, informed consent will be obtained from the parent or from the adolescents if they are 18 years or older. Adolescents under 18 years will also need to provide assent to participate. All study procedures have received approval from the Drexel University Institutional Review Board, under protocol number 2401010341. All participants will be compensated up to US $275 for their participation (see the “Ecological Momentary Assessment” section for more details). Data will be stored securely on Drexel’s encrypted servers, and analytic datasets will be stripped of identifiers.

### Participants

We will enroll 70 adolescent girls with clinically significant binge eating (ie, engaging in at least 1 binge eating episode per week over the past 12 weeks). Both subjective and objective binge episodes will be counted given literature demonstrating that the feeling of loss of control, rather than the size of the binge episode, is more closely linked to ED severity [40,41]. Thus, to be included in the study, participants will need to be between 14 and 19 years old; be able to speak, read, and write English; have a smartphone they would be willing to complete daily surveys on for the course of the study; and were assigned female at birth. Participants will be excluded if they are currently receiving treatment for binge eating and/or weight loss or if they will be starting treatment during the course of the study, if they have begun a course of or changed the dosage of any medication known to affect appetite or body composition in the past 3 months, if they are currently pregnant or have plans to become pregnant, or if they have a developmental disability (eg, autism spectrum disorder) or are currently experiencing severe psychopathology that would limit their ability to engage in the study or would otherwise pose a risk to their safety (eg, severe depression, substance dependence, active psychotic episode, imminent suicide risk). The final 2 exclusion criteria will be assessed in consultation with a licensed clinical psychologist. Although such criteria may exclude certain individuals who represent the broader clinical population, these criteria are in place to ensure participant safety during this observational study.

### Study Procedures

#### Recruitment and Screening

Participants will be recruited nationwide through online advertising (eg, on social media platforms) and through a registry of adolescents who are either waitlisted or in the screening process for participation in a clinical trial for binge-spectrum EDs at Drexel University. Participants who appear to be eligible following the screening process and are interested in participating will be invited to a 2-hour baseline assessment with study staff held either virtually via Zoom or in person.

#### Baseline Measures

Participant characteristics, namely race, ethnicity, age, socioeconomic status, year in school, and history of ED diagnoses and treatment receipt, will be measured at baseline through a demographics survey.

Diagnostic status will be established at baseline using the binge and compensatory modules from the eating disorder examination [42], a well-validated interview used to evaluate ED pathology, which will be administered by trained study staff. Participants who report 12 or more binge episodes but fewer than 12 compensatory behaviors will be classified as having BED, whereas those with 12 or more compensatory behaviors will be classified as having BN. If participants report at least 12 objectively large episodes, they will be classified as “full-threshold,” whereas those with fewer objectively large episodes will be classified as “sub-threshold” [43].

Body concern will be measured at baseline using the Body Shape Questionnaire [44], a 34-item self-report questionnaire. Items inquire about the frequency of concerns related to one’s body and are rated on a 6-point scale ranging from 1 (“Never”) to 6 (“Always”). A total score will be calculated by summing all of the items.

Body checking will be measured at baseline using the Body Checking Questionnaire [45], a 23-item self-report measure. Items inquire about the frequency of various body checking behaviors and are rated on a 5-point scale ranging from 1 (“Never”) to 5 (“Very often”). The Body Checking Questionnaire contains 3 subscales that assess body checking related to overall appearance, specific body parts, and idiosyncratic rituals. A total score will be calculated by summing all of the subscale items.

ED pathology will be measured at baseline using the Eating Disorder Examination Questionnaire [46], a 28-item self-report questionnaire. Items pertaining to frequency are rated on a 7-point scale ranging from 0 (“No days”) to 6 (“Every day”); items pertaining to distress are rated on a 7-point scale ranging from 0 (“Not at all”) to 6 (“Markedly”). The Eating Disorder Examination Questionnaire contains 4 subscales measuring eating concern, weight concern, shape concern, and dietary restraint. A global score will be calculated by taking the mean of all the subscale scores.

#### Ecological Momentary Assessment

EMA surveys will be facilitated by the EMA app Avicenna. Following the completion of the interview and self-report measurements at the baseline visit, research staff will assist with installing the EMA app and how to use it. During the 3-week EMA protocol, signal-contingent (ie, participants are prompted to complete surveys at semirandom times) and event-contingent (ie, participants will be instructed to initiate surveys when they engage in binge eating or a compensatory behavior if it occurs outside signal-contingent survey windows) will be used. The timing of the first and last EMA surveys will be tailored to participants’ self-reported weekday or weekend bedtimes and wake times such that they arrive at random times within 1 hour of waking up and within 1 hour of going to bed. The remaining 3 surveys will come between those windows, excluding hours during which adolescents are in school. To promote high compliance, participants will complete a test day (“day zero”) and will be provided with feedback from a research assistant. Then, participants will receive 5 signal-contingent surveys a day for 21 days, for a total of 105 surveys. Surveys will pertain to engagement in body checking and both cognitive and behavioral symptoms of EDs (see questions below). Participants will earn US $2.14 for each completed survey and earn a US $50 bonus if they complete 85% or more surveys for a maximum possible compensation of US $275. To promote high response rates, participants will receive personalized reminder texts coupled with memes and GIFs throughout the EMA period [47]. Although such engagement strategies may have unintended effects on responses, at least 1 EMA study with adolescents revealed that such strategies appear to increase compliance without increasing careless responding [48].

Body checking will be measured using the same set of questions included in previous EMA studies [16,17] and will require participants to report the number of times they had engaged in the following behaviors since the last survey: (1) weighing oneself, (2) feeling thighs for fatness or checking to see if thighs spread while sitting down, (3) sucking in the stomach, (4) feeling or pinching stomach to measure fatness, (5) comparing one’s body to other individuals, (6) checking body size in the mirror, and (7) checking for fat jiggling. In aim 1 (characterizing body checking), engagement in specific forms of body checking will be examined descriptively. In aim 2 (prospective associations between body checking and ED symptoms), body checking will be collapsed and treated as a binary variable representing engagement in *any* form of body checking at each survey. In aim 3 (body checking reactivity during EMA), body checking will be defined as a count variable representing the number of times each participant engaged in *any* form of body checking in a given day.

Body satisfaction will be measured through a single question: “Right now, how satisfied do you feel with your body shape or weight?” Responses will be rated on a Likert scale ranging from 1 (“Not at all satisfied”) to 5 (“Very satisfied”). Body satisfaction will be treated as a continuous variable representing current degree of body satisfaction at each survey.

Fear of weight gain will be measured through a single question: “To what extent are you currently afraid of gaining weight?” Responses will be rated on a Likert scale ranging from 1 (“Not at all afraid”) to 5 (“Very afraid”). Fear of weight gain will be treated as a continuous variable representing current degree of fear of weight gain at each survey.

Binge eating will be measured by first asking whether participants have eaten since the last survey. If so, participants will answer “While you were eating, did you feel a sense of loss of control (eg, like a car without brakes that could not stop)?” on a Likert scale ranging from 1 (“Not at all”) to 5 (“Extremely”). Consistent with previous EMA studies, participants were counted as having endorsed loss-of-control eating if they reported a “3” or higher on either question at least once during the recording period [49]. Binge eating will be treated as a binary variable representing whether a binge eating episode occurred at each survey.

Dietary restraint will be measured by asking, “Since the last survey, to what extent have you attempted each of the following, even if you were not successful?” Answer choices included (1) “Tried to limit the amount you ate,” (2) “Tried to avoid eating certain foods that you like,” and (3) “Tried to delay eating.” Responses will be rated on a Likert scale ranging from 1 (“Did not attempt”) to 5 (“Attempted as hard as I could”). Dietary restraint will be treated as a binary variable representing a response of 3 (“Moderately attempted”) or higher on any of the dietary restraint choices.

Dietary restriction will be measured by asking, “Since the last survey, were you successful in actually _______ in order to influence your shape and/or weight?” where the blank field will be replaced with (1) “limiting the amount that you ate,” (2) “avoiding eating certain foods that you like,” and (3) “trying to delay eating.” Responses will be in a “Yes/No” format. Dietary restriction will be treated as a binary variable representing engagement in *any* form of dietary restriction at each survey.

Compensatory behaviors will be measured by asking, “Since the last survey, have you engaged in any of the following? Select all that apply.” Options will include (1) “self-induced vomiting,” (2) “taking laxatives to influence your shape and/or weight,” (3) “taking diet pills to influence your shape and/or weight,” (4) “taking diuretics to influence your shape and/or weight,” (5) “exercise to influence your shape and/or weight,” and (6) “other behavior to influence your shape and/or weight.” Compensatory behaviors will be collapsed and treated as a binary variable representing engagement in *any* compensatory behaviors at each survey.

Compliance will be calculated as the number of completed signal-contingent EMA surveys out of 105 possible EMA surveys. To gage engagement, we will additionally report the total number of completed signal-contingent and event-contingent surveys.

### Statistical Analysis

#### Sample Size Determination and Data Exclusions

Sample size was determined through power curve analyses in R version 4.2.3 [50] using the package *EMATools* [51], which indicated that to detect a medium-sized effect with 80% power using mixed-effects models, we would need to recruit a minimum of 70 participants. To the extent that study resources (eg, budget, staffing) permit, more participants will be recruited to account for poor compliance and/or dropouts.

The data from the test day will be excluded from analyses. To promote high survey compliance, participants who achieve less than 50% compliance on the test day will receive a warning message. Participants who achieve less than 50% compliance on the test day and the first day of the study will be removed from the study.

#### Study Aims

Aim 1 of this study is to characterize body checking among adolescents with binge-spectrum EDs. To evaluate aim 1, descriptive statistics (ie, means and SDs) will be calculated for the frequency of each type of body checking behavior and the percentage of participants who endorsed each type of behavior over the EMA period, both in the full sample and by diagnostic category (BED-spectrum vs BN-spectrum), diagnostic threshold (full-threshold vs subthreshold), racial group, and gender identity. Additionally, the distribution of body checking frequency will be examined to capture when body checking behaviors most frequently occur throughout the day.

Aim 2 of the study is to assess whether frequency in body checking at 1 observation is associated with other ED symptoms at the same observation and at the next observation. To preserve analytic power, we plan to define body checking frequency as a composite binary variable representing the occurrence of body checking that aggregates counts across all types of body checking behaviors. However, it is possible that body checking (as a composite variable) may be reported so frequently that it fails to have any predictive ability or that certain body checking behaviors may have stronger prospective effects than others. Thus, we will conduct sensitivity analyses that examine each form of body checking separately as opposed to relying on a composite variable. We will use linear mixed-effects models for continuous outcomes (ie, ratings of body dissatisfaction and fear of weight gain) and generalized mixed-effects models using a binomial distribution with a logit link function for binary outcomes (ie, binge eating, compensatory behaviors, dietary restraint, and dietary restriction). All models will include body checking as a fixed predictor and a random intercept per participant. Consistent with other studies [52], models assessing binge eating and dietary restraint or restriction will control for binge eating and dietary restraint or restriction, respectively, at the previous observation. False discovery rate corrections will be used to correct for multiple comparisons.

Aim 3 is to assess whether body checking behaviors are reactive to EMA, such that they decrease over the course of the EMA period. To evaluate aim 3, we will first create a composite count score that aggregates frequencies of all body checking behaviors per person per day. Then, we will fit a generalized linear mixed model with a Poisson distribution to examine whether the count of body checking behaviors decreases as a function of time. We will include a fixed effect of time (ie, day of study) and a random intercept per subject. If the model yields poor fit (ie, there is over- or underdispersion), then a Quasi-Poisson will be used, and data will be examined for both zero-truncation and zero-inflation. To examine differences in body checking counts between the start and end of study participation, we will conduct a Poisson regression to examine associations between counts on day 1 to 3 and day 19 to 21. We elected to examine the first and last 3 days to obtain a more stable metric of frequency. Additionally, we will regress body checking behaviors across 2 time windows, day 1 to day 10 and day 11 to day 21. Given that the frequency of body checking may be directly associated with survey compliance, we will examine whether body checking frequency is correlated with the number of surveys completed. Further, in our Poisson regression model, we will control for the frequency of surveys completed to examine the rate of reduction in body checking behaviors in the first and second halves of the study period.

## Results

Recruitment began in January 2025, and data collection is expected to conclude in March 2026. Data analyses and dissemination of results will be completed by August 2026.

## Discussion

### Study Implications and Future Directions

To date, there is limited literature on the role of body checking in maintaining binge-spectrum EDs among adolescents [12,14,23,53]. Thus, this study will provide valuable insights into the types, frequencies, and effects of body checking behaviors on ED symptoms among adolescents with binge-spectrum EDs. The findings from this study will clarify whether body checking behaviors are prospectively associated with engagement in other ED symptoms and may provide direction for research on mediators and moderators of this relationship, which is critical for better understanding maintaining mechanisms of EDs in adolescents. Furthermore, this study will evaluate whether body checking shows reductions when subject to self-monitoring during EMA. If our hypotheses are supported and body checking behaviors decrease along with other ED symptoms, this study could inform the importance of formally targeting body checking during treatments, such as CBT-E, and could inform clinicians’ decisions around which symptoms to prioritize. Further, the findings from this study may provide a stepping stone for the development of scalable, stand-alone interventions to reduce body checking behaviors. Although such stand-alone interventions will not replace gold-standard approaches such as CBT-E, they represent a low-cost, accessible option for individuals with EDs that could be used as an adjunct to available treatments or a brief intervention while patients await the receipt of more comprehensive treatment. Digital self-monitoring could also have high utility as a tool for ED prevention, given literature which supports that higher engagement in body checking in adolescents is longitudinally linked to the development of ED symptoms [24]. Future research should aim to pilot such digital interventions to establish preliminary acceptability and feasibility.

### Strengths and Limitations

The strengths of this study include the use of a transdiagnostic sample of adolescents with binge-spectrum EDs, which will improve the generalizability of the results. Furthermore, the inclusion of a broad range of body checking behaviors, as opposed to assessing body checking with 1 or 2 items as past literature has done [20], will provide critical insights into the frequency and timing of these behaviors among adolescents.

The limitations of this study include the lack of follow-up assessments, which make it difficult to assess reactive effects to self-monitoring, whether body checking continues to decrease, plateaus, or eventually increases back to baseline. Likewise, given that adherence to self-monitoring may have a dose-response relationship with reactive reductions in body checking, it is possible that the incentives used to increase compliance (ie, financial compensation and personalized reminder texts with memes and GIFs) may confound aim 3. Given that incentives are needed to promote compliance, otherwise data missingness would preclude the detection of hypothesized relationships, future studies might consider varying sampling procedures (eg, fewer surveys per day) to more carefully test for a dose-response relationship between self-monitoring via EMA and body checking. Future work should examine these behaviors over longer periods and include follow-up assessments to better understand how effectively self-monitoring intervenes on body checking and how decreases in body checking may correlate with reductions in other indices of ED pathology. Of note, given the EMA design of this study, it is possible that reductions in other ED behaviors may be driven by self-monitoring or may arise from reductions in the overvaluation of shape and weight, as manifest by body checking. This study is further limited by its exclusion of boys. Given that boys use a different set of methods to body check [39], future work should use questionnaires, such as the Male Body Checking Questionnaire, to assess body checking in boys [38]. Likewise, given heterogeneity in body image ideals across cultures [54], it is possible measures used in this study will not appropriately capture body checking behaviors, and caution is warranted when examining racial or ethnic differences in body checking. Finally, although we used EMA items reported in other studies [16,17,52,55], including those with adolescents, some of these items have yet to undergo proper validation efforts, particularly for administration among adolescents.

### Conclusion

In conclusion, this study will elucidate the nature and frequency of body checking, body checking’s prospective relations to ED symptoms, and the potential reactivity of body checking to digital self-monitoring (via EMA) among adolescent girls with binge-spectrum EDs. The data afforded by this study will be critical for understanding the presentations of body checking in adolescents’ daily lives and how it may maintain binge-spectrum EDs. This study will also inform optimal strategies for intervening on body checking and improving long-term ED treatment outcomes in adolescents with EDs.

## Abbreviations

BED: binge eating disorder
BN: bulimia nervosa
CBT: cognitive behavioral therapy
CBT-E: enhanced cognitive behavior therapy
ED: eating disorder
EMA: ecological momentary assessment
